# Production of Siderophores Increases Resistance to Fusaric Acid in *Pseudomonas protegens* Pf-5

**DOI:** 10.1371/journal.pone.0117040

**Published:** 2015-01-08

**Authors:** Jimena A. Ruiz, Evangelina M. Bernar, Kirsten Jung

**Affiliations:** 1 Instituto de Investigaciones en Biociencias Agrícolas y Ambientales, Consejo Nacional de Investigaciones Científicas y Técnicas, Facultad de Agronomía, Universidad de Buenos Aires, Ciudad Autónoma de Buenos Aires, Argentina; 2 Ludwig-Maximilians-Universität München, Munich Center for integrated Protein Science (CiPSM) at the Department of Biology I, Microbiology, Martinsried, Germany; 3 Departamento de Química Biológica, Facultad de Ciencias Exactas y Naturales, Universidad de Buenos Aires, Ciudad Autónoma de Buenos Aires, Argentina; Case Western Reserve University, UNITED STATES

## Abstract

Fusaric acid is produced by pathogenic fungi of the genus *Fusarium*, and is toxic to plants and rhizobacteria. Many fluorescent pseudomonads can prevent wilt diseases caused by these fungi. This study was undertaken to evaluate the effect of fusaric acid on *P. protegens* Pf-5 and elucidate the mechanisms that enable the bacterium to survive in the presence of the mycotoxin. The results confirm that fusaric acid negatively affects growth and motility of *P. protegens*. Moreover, a notable increase in secretion of the siderophore pyoverdine was observed when *P. protegens* was grown in the presence of fusaric acid. Concomitantly, levels of enzymes involved in the biosynthesis of pyoverdine and enantio-pyochelin, the second siderophore encoded by *P. protegens*, increased markedly. Moreover, while similar levels of resistance to fusaric acid were observed for *P. protegens* mutants unable to synthesize either pyoverdine or enanto-pyochelin and the wild type strain, a double mutant unable to synthesize both kinds of siderophores showed a dramatically reduced resistance to this compound. This reduced resistance was not observed when this mutant was grown under conditions of iron excess. Spectrophotometric titrations revealed that fusaric acid binds not only Fe^2+^ and Fe^3+^, but also Zn^2+^, Mn^2+^ and Cu^2+^, with high affinity. Our results demonstrate that iron sequestration accounts at least in part for the deleterious effect of the mycotoxin on *P. protegens*.

## Introduction

Fusaric acid (FA, 5-butylpyridine-2-carboxylic acid) [1] is a fungal metabolite produced by several members of the genus *Fusarium* that contribute to wilt and rot diseases of plants [1, 2, 3], including crop species belonging to the families *Gramineae*, *Leguminosae*, *Alliaceae and Solanaceae*, which result in significant economic losses worldwide [4, 5, 6, 7, 8, 9]. Currently, application of synthetic fungicides is the strategy most frequently used to control diseases caused by *Fusarium* species. However, in light of the environmental effects of synthetic pesticides and the emergence of fungicide-resistant strains, the use of bacterial species as biocontrol agents is considered to be a worthwhile and promising alternative.

Several studies have demonstrated that fluorescent *Pseudomonas* spp. can efficiently colonize roots infected by *Fusarium oxysporum* [10] and suppress plant diseases caused by this fungus [11, 12, 13]. However, factors such as the physicochemical properties of the soil and the specific interactions that take place between plants, fungi and bacteria are known to modulate the efficacy of biocontrol of soil-borne pathogens [14]. Thus, it has been demonstrated that FA limits production of the antifungal metabolite phenazine-1-carboxamide by *P. chlororaphis* [15, 16] and represses expression of genes involved in the synthesis of the polyketide antibiotic 2,4-diacetylphoroglucinol (DAPG) in *P. fluorescens*, both *in vitro* and *in vivo* [12, 17] under conditions that normally favor antibiotic biosynthesis by fluorescent *Pseudomonas* spp. This suggests that FA reduces the ability of these *Pseudomonas* strains to compete against pathogens in soil. In addition, FA is highly toxic to several plant and bacterial species [1, 2, 3, 18], but the basis for this toxicity is not clearly understood.

Overall, little is known about the negative effects of FA on fluorescent *Pseudomonas* spp. or the mechanisms that enable these bacteria to survive in its presence. In this work, we investigate the effects of FA on growth, motility, biofilm formation and pyoverdine synthesis in the well-known biocontrol strain *Pseudomonas protegens* Pf-5 (former *P. fluorescens* Pf-5), and identify differences in the protein composition of subcellular fractions of cells grown in presence or absence of FA, which allowed us to elucidate the mechanisms that contribute to the survival of *P. protegens* in the presence of the mycotoxin. The results show that, under iron-limited conditions, FA negatively affects growth of *P. protegens* by sequestering iron, thus making this essential nutrient unavailable to the bacterium. Accordingly, siderophores promote survival of *P. protegens* Pf-5 in the presence of FA, suggesting their important role in the biocontrol of *Fusarium* spp. that produce the toxin.

## Experimental Procedures

### Bacterial strains, plasmids and culture conditions

The strains and plasmids used in this study are listed in Table 1.

**Table 1:** Bacterial strains and plasmids used in this study.

**Strain or plasmid**	**Relevant genotype or description**	**Reference or source**
***E. coli*** **strains**		
MG1655	F^-^ λ^-^ *ilvG rfb50 rph-1*	[60]
S17–1	*recA pro hsdR* RP4–2-Tc::Mu-Km::*Tn7*	[61]
JM109	*endA1 glnV44 thi-1 relA1 gyrA96 recA1 mcrB* ^+^ Δ(*lac-proAB*) e14- [F’ *traD36 proAB* ^+^ *lacI* ^q^ *lacZΔM15*] *hsdR17*(r_K_^-^m_K_^+^)	[62]
DH5α-λpir	F^-^ *endA1 glnV44 thi-1 recA1 relA1 gyrA96 deoR nupG* Φ80d*lacZ*ΔM15 Δ(*lacZYA-argF*)U169, *hsdR17*(r_K_^-^ m_K_^+^)*/λpir*	[63]
WM3064	*thrB1004 pro thi rpsL hsdS lacZ*ΔM15 RP4-1360 Δ(*araBAD*)*567* Δ*dapA1341*::[*erm pir*(wt)	W. Metcalf, University of Illinois, Urbana-Champaign
***P. protegens*** **strains**		
Pf-5	Wild type; plant commensal isolated from the rhizosphere of cotton	[64]
Pf-21	Pf-5 Δ*pvdF;* unable to produce pyoverdine	This study
Pf-4	Pf-5 Δ*pchF*; unable to produce pyochelin	This study
Pf-21.10	Pf-5 Δ*pvdF ΔpchF*; unable to produce pyoverdine and pyochelin	This study
Pf-5fa	Pf-5 Δ(PFL_1003-PFL_1006) Km^r^	This study
Pf-5fb	Pf-5 Δ(PFL_0155-PFL_0159) Km^r^	This study
Pf-5fab	Pf-5 Δ(PFL_1003-PFL_1006-PFL_0155-PFL_0159) Km^r^	This study
**Plasmids**		
pNPTS138-R6KT	*mobRP4 ori*-R6K *sacB*; suicide plasmid for in-frame deletions; Km^r^	[21]
pNPTS*pvdF*	*pvdF* deletion fragment in pNPTS138-R6KT	This study
pNPTS*pchF*	*pchF* deletion fragment in pNPTS138-R6KT	This study
pEX18Tc	Tc^r^, *oriT* ^+^, *sacB* ^+^, gene replacement vector	[19]
pETcfa	Δ(PFL_1003-PFL_1006) fragment in pEX18Tc	This study
pETcfb	Δ(PFL_0155-PFL_0159) fragment in pEX18Tc	This study
pFLP2	Ap^r^, oriT^+^, sacB^+^, contains *Flp* recombinase from *S. cerevisiae*	[19]

Deletion mutants of *P. protegens* Pf-5 were constructed by gene replacement using the suicide plasmid pEX18Tc [19] followed by Flp-mediated marker excision as previously described [20], or the suicide plasmid pNPTS138-R6KT [21]. Information on primers used to construct the upstream, downstream and overlapped DNA fragments are available on request. All gene replacement mutants were checked by PCR and sequencing.

*P. protegens* strains were cultured under aeration at 30°C in either LB, King´s medium B broth (KMB) or minimal E_2_ medium [22] supplemented with 1 mM MgSO_4_, 0.1% (vol/vol) trace-metal (MT) solution [22], and glucose at a final concentration of 0.6% (wt/vol). *E. coli* strains were routinely grown at 37°C in LB medium. Where necessary, media were solidified by adding 1.5% (wt/vol) agar. Kanamycin, tetracycline and carbenicillin were used at concentrations of 50 μg ml^-1^, 10 μg ml^-1^ and 200 μg ml^-1^, respectively. When required, cultures were supplemented with different concentrations of FA (Sigma, St. Louis, Mo.) from a 280 mM stock solution prepared by dissolving the compound in 18% (vol/vol) methanol and adjusting the pH of the solution to 6.5 with 2 N NaOH.

To evaluate the effect of FA on growth or to prepare protein extracts, cells from an overnight culture grown in glucose E_2_ medium were inoculated into fresh medium (with or without FA) to give an initial optical density at 600 nm (OD_600_) of 0.05. Cultures were grown in shaken Erlenmeyer flasks or 96-well microtiter plates at 30°C. The minimal inhibitory concentration (MIC) was determined as the concentration of the compound that completely inhibited growth.

### Molecular biological techniques

Plasmid and genomic DNAs were isolated using HiYield plasmid minikits (Sued-Laborbedarf Gauting) and DNeasy blood and tissue kits (Qiagen), respectively. DNA fragments were purified from agarose gels using the Hi-Yield PCR cleanup and gel extraction kit (Sued-Laborbedarf). Phusion high-fidelity DNA polymerase (Finnzymes) was used according to the supplier’s instructions. Restriction enzymes were purchased from New England Biolabs and used according to the manufacturer’s directions.

### Pyoverdine quantification

Pyoverdine in the supernatants of cultures grown for 16 h at 30°C and 1000 rpm in 96-well microtiter plates containing glucose E_2_ medium was quantified with an Infinite 500 Plate Reader (Tecan, Crailsheim) by measuring fluorescence emission at 485 nm after excitation at 420 nm. The values were expressed relative to the OD_600_ of the corresponding cultures.

### Motility assays

*P. protegens* Pf-5 was cultured overnight in KMB [23] at 30°C. Swimming and swarming motility was evaluated using the protocol previously described by Gross *et al*. [24] with slight modifications. Briefly, 0.005 mL of the culture was spotted at the center of five replicate plates containing 20-fold diluted KMB with or without FA, and 0.3% and 0.6% (wt/vol) Bacto-Agar (Difco) for the evaluation of swimming motility and determination of swarming motility, respectively. Plates were incubated overnight at 25°C. The experiment was repeated three times.

### Biofilm formation

*P. protegens* Pf-5 cells were grown overnight in glucose E_2_ medium, and an aliquot of this culture was inoculated into the wells of a polystyrene 96-well plate, each containing 0.150 mL of fresh medium with or without FA, to give an initial OD_600_ of 0.05. Plates were incubated for 24 h at 30°C in a humid chamber. After incubation, the OD_600_ of non-attached (planktonic) cells was measured, and these cells were then removed by rinsing the plate thoroughly with water. The biomass of attached cells (biofilm) was quantified by staining with crystal violet and subsequent determination of the absorbance at 595 nm (A_595_), according to the protocol of Merritt *et al*. [25]. These values were normalized to the biomass (OD_600_) of non-attached bacteria. The assay was performed three times with three independent cultures each time and ten replicates of each culture.

### Protein electrophoresis and MALDI-TOF

Six independent cultures of *P. protegens* Pf-5 with an initial OD_600_ of 0.05 were incubated aerobically at 30°C in 400 mL of glucose E_2_ medium with or without 2 mM FA. After 12 h, cells were exposed to 1 mg mL^-1^ chloramphenicol to inhibit protein synthesis, harvested at 4°C and washed with ice-cold wash buffer (10 mM Tris-HCl, pH 7.5; 0.1 mg mL^−1^ chloramphenicol). The cell pellet was frozen in liquid nitrogen and stored at -80°C until further use. To prepare cytosolic and total membrane proteins, cells were resuspended in ice-cold disruption buffer (10 mM Tris-HCl, pH 7.5; 50 μg/mL RNase; 50 μg/mL DNase; 100 μg/mL lysozyme; 1 mM PMSF) and disrupted by sonication on ice (ten 30-s pulses administered at intervals of 30 s). Cell debris was removed by three centrifugations for 10 min at 16,100×g at 4°C and the supernatant was transferred to an ultracentrifuge tube. The crude extract was fractionated by ultracentrifugation (99,000×g for 60 min at 4°C) to obtain the cytosolic and membrane fractions.

Proteins of the supernatant (cytosolic fraction) were analyzed by two-dimensional gel electrophoresis, as follows. An aliquot containing 1 mg of protein in all was lyophilized overnight, suspended in rehydration buffer (8 M urea; 2 M thiourea; 2% (wt/vol) CHAPS; 1.25% (wt/vol) IPG buffer pH3–10 (GE Healthcare, Munich); 28.4 mM DTT; bromophenol blue) and first fractionated on IPG strips (pH 3–10, 24 cm, linear; GE Healthcare) at 40 kVh. The strips were then equilibrated twice for 15 min each in buffer (50 mM Tris-HCl pH 6.8; 6 M urea; 30% (vol/vol) glycerol; 4% (wt/vol) SDS; 18.2 mM DTT), before fractionation in the second dimension was performed by SDS-PAGE [26] using 1-mm thick gels (25.5 × 19.6 cm) with 13% acrylamide in the separation gel and 7% in the spacer gel. Gels were run at 15°C at 150 mA. After electrophoresis, gels were stained for 1 h in Coomassie blue solution [40% (vol/vol) ethanol; 10% (vol/vol) acetic acid; 0.2% (wt/vol) Coomassie brilliant blue R250)], destained for 1 h with destaining solution [40% (vol/vol) ethanol; 10% (vol/vol) acetate] and finally destained with 10% (vol/vol) acetic acid [27]. Finally, gels were scanned and analyzed with PDQuest software (Bio-Rad, Munich).

The pellet obtained after ultracentrifugation, corresponding to the total membrane fraction, was washed with ice-cold 1 mM Tris-HCl/0.5 mM PMSF, and subjected to ultracentrifugation (99,000×g for 60 min at 4°C). The pelleted membranes were then resuspended in 0.5 mL of TG buffer (50 mM Tris-HCl, 10% (vol/vol) glycerol). The membrane proteins were quantified as described by Peterson [28]. An aliquot of each sample containing 100 μg of protein was solubilized in SDS sample buffer (125 mM Tris-HCl pH 6.8, 22% (vol/vol) glycerol, 4% (wt/vol) SDS, 0.05% (wt/vol) bromophenol blue and 0.065 mM DTT), incubated for 2 h at room temperature and fractionated by (SDS-PAGE) [26] on 20 × 20 cm gels with 7.5% or 12% acrylamide in the separation gel and 4% acrylamide in the spacer gel. Gels were run overnight at 4°C and 85 V. After electrophoresis, gels were stained as described above. Proteins expressed at visibly different levels in cells exposed or not to FA were identified by peptide fingerprint analysis [29] using a matrix-assisted laser desorption ionization–time of flight (MALDI-TOF) mass spectrometry (MS) system (Voyager DE STR; Applied Biosystems). Samples were prepared and identified as described previously [30].

### Determination of chelate formation by spectrophotometric titration

FA was diluted in double-distilled (Milli-Q) water at a final concentration of 0.14 mM from a stock solution of 280 mM prepared as described above. UV absorption spectra of FA alone and FA incubated with increasing amounts of Fe^2+^, Fe^3+^, Cu^2+^, Mn^2+^ and Zn^2+^ were analyzed spectrophotometrically (Ultrospec 2100 pro, GE Healthcare) over the wavelength interval between 200 and 300 nm. Stock solutions of the corresponding metals were freshly prepared at a concentration of 0.01M in 0.01 N HCl, mixed with FA and incubated for 2 min prior to measurement. Fusaric acid shows maximum absorption at 270 nm (A_275_), and peak height is affected by metal binding. The absorption intensity measured at 270 nm was therefore used to calculate the apparent affinity constant (K_0.5_) of FA for the different metals, which corresponds to the metal concentration that causes a half-maximal change in absorption at 270 nm. For this purpose, the data were fitted with the SigmaPlot program (Jandel Scientific) using the following equation: ΔA_275_/A_275_ = (ΔA_275_/A_275max_)x[M]/(K_0.5_+[M]), where ΔA_275_ is the change in absorption observed upon addition of metals, A_275_ is the initial absorption of FA in the absence of metals, ΔA_275_/A_275max_ corresponds to the maximum absorption change produced by the metal, and [M] is the metal concentration. All measurements were performed at least three times.

### Extraction and purification of enantio-pyochelin

Enantio-pyochelin (EPch) was extracted from *P. protegens* Pf-21 and purified by HPLC on a Thermo-Scientific Dionex UltiMate 3000 Series HPLC system and a Hypersil Gold C18 preparative column (250×10mm, 5 μm; Thermo Scientific) following the protocol described by Youard *et al*. [31]. Aliquots of 0.05 mL were injected into the system and fractionated at room temperature using an isocratic gradient of 28.5% (vol/vol) acetonitrile + 0.1% (vol/vol) trifluoroacetic acid at a flow rate of 1mL/min. The chromatographic profile of the extract from *P. protegens* Pf-21 was similar to that reported by Youard *et al*. [31] and was compared to the chromatographic profile of an extract from the *P. protegens* mutant (Pf-21.10), which is unable to produce EPch. As a result, a peak with a retention time of 17 min with maximal absorbance at 235 nm was identified as EPch and retained. EPch concentration was determined using the molar extinction coefficient (ε_315nm_: 4200) [32]. The biological activity of EPch was tested in cultures of the siderophore-negative mutant *P. protegens* Pf-21.10.

### Quantification of FA in the supernatants by HPLC

Cultures of P. *protegens* Pf-5 and *E. coli* MG1655 were grown aerobically overnight in glucose E_2_ medium, centrifuged at 4°C for 15 min at 16.100×g and filtered through a pore size of 0.22 μM. The FA content in the supernatant was quantified by HPLC using the protocol described by Notz *et al*. [17] using a Thermo Scientific Dionex UltiMate 3000 Series HPLC system equipped with a C18-Silica column (150×2mm, 3 μm, Grace, Worms, Germany). The samples were quantified using a calibration curve of known FA concentrations (SigmaA St. Louis, Mo.).

## Results

### FA affects growth, motility and biofilm formation in *P. protegens* Pf-5

**Figure 1:**
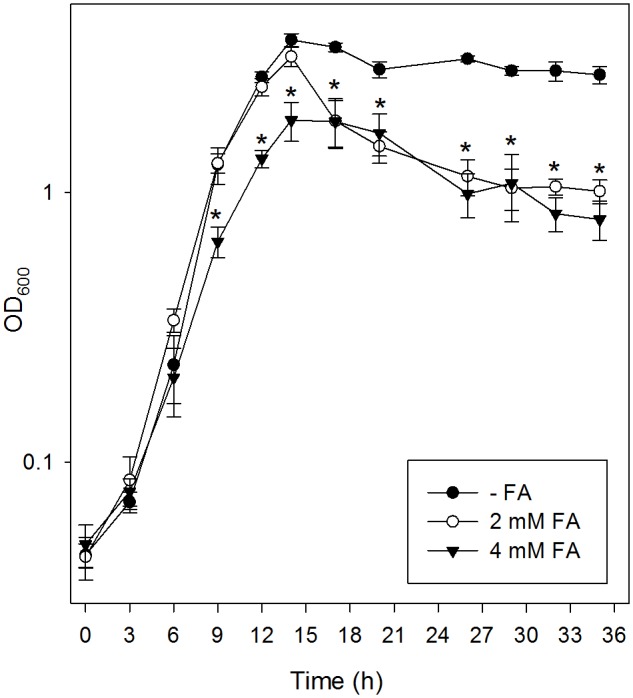
Effect of FA on growth of *P. protegens* Pf-5. Cultures of *P. protegens* Pf-5 were grown aerobically in E_2_ glucose minimal medium supplemented or not with the indicated concentrations of FA. Growth was monitored by measurement of the OD_600_ at the indicated times after inoculation. OD_600_ levels are mean values for three independently grown cultures, and error bars depict standard deviations of the mean. The asterisk (*) denotes significant differences from the culture grown without FA (P<0.05) using ANOVA.

We first determined the impact of different concentrations of FA on the growth of *P. protegens* Pf-5 in glucose E_2_ minimal medium. Growth was completely inhibited when the medium contained FA concentrations ≥7 mM. Subsequently, we analyzed the effects of two sub-inhibitory concentrations: use of 4 mM FA caused a reduction in the growth rate, and cell death and lysis were observed after the entry into stationary phase of growth in the presence of either 2 or 4 mM FA (Fig. 1).

**Figure 2:**
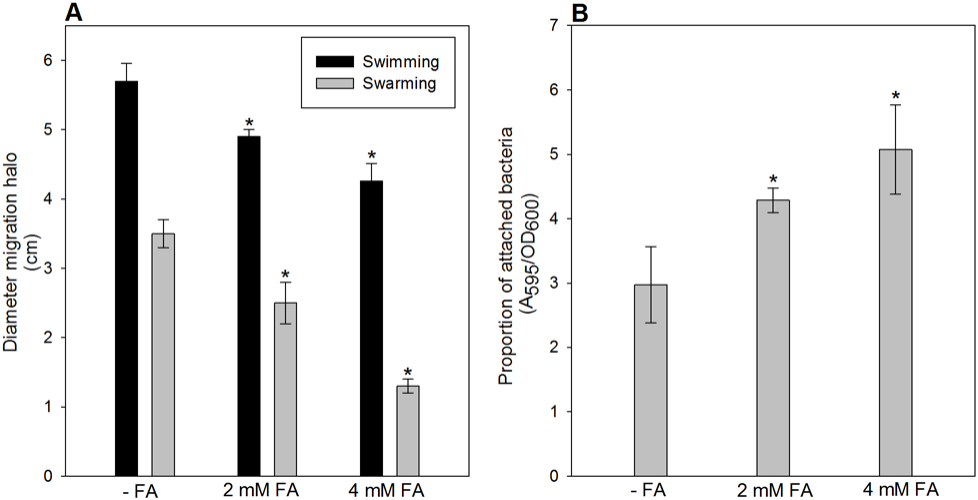
Effect of FA on cell motility and biofilm formation by *P. protegens* Pf-5. **A**. Cultures of *P. protegens* Pf-5 (wild type) were grown aerobically overnight to OD = 3 in KMB medium. An aliquot was spotted onto the centre of plates containing 20 fold diluted KMB medium supplemented or not with FA and solidified with various concentrations of Bacto-Agar, depending on which motility was to be evaluated. After 24 h of incubation at room temperature, the distance of the migration front from the point of inoculation was measured. **B**. Cultures of *P. protegens* Pf-5 were grown in 96-well polystyrene plates containing E_2_ glucose minimal medium supplemented or not with FA. After 24 h of incubation, biofilm formation was assessed by the determining the proportion of attached and non-attached bacteria (A_595_/OD_600_). In all cases, error bars indicate the standard deviation of the mean. The asterisk (*) denotes significant differences (P<0.05) using ANOVA.

As motility and the ability to form biofilms are important for colonization of plant roots, survival in the rhizosphere and biocontrol of soil-borne pathogens [33, 34, 35], the effects of sub-inhibitory concentrations of FA on swimming and swarming motility, as well as the ability to adhere to a polystyrene surface (a proxy for biofilm formation) was analyzed. Fig. 2A shows that addition of FA significantly reduced swimming and swarming motility of *P. protegens* Pf-5. Conversely, the presence of FA in the growth medium had a positive effect on biofilm formation (Fig. 2B). Both reduction of bacterial motility and enhancement of biofilm formation were positively correlated with the concentration of FA present in the medium (Fig. 2A and B).

### FA induces the expression of proteins involved in iron acquisition

As mentioned above, *P. protegens* Pf-5 is able to survive in the presence of FA concentrations lower than 7 mM. Clusters of genes involved in resistance to and detoxification of FA (*fus* genes) have been identified in both *Burkholderia cepacia* and *Klebsiella oxytoca* [36, 37], and the proteins encoded by *fus* genes show homology to multidrug efflux transporter systems involved in resistance to antibiotics, biocides, dyes, detergents and molecules involved in cell-cell communication [38]. *P. protegens* Pf-5 possesses two clusters of genes (PFL_1003 to PFL_1006 and PFL_0154 to PFL_0159) that code for products with high similarity to proteins involved in rendering *B. cepacia* and *K. oxytoca* resistant to FA. However, elimination of both gene clusters did not alter the level of resistance to FA exhibited by *P. protegens* Pf-5. The MIC obtained for *P. protegens* Pf-5, *P. protegens* Pf-5fa, *P. protegens* Pf-5fb and *P. protegens* Pf-5fab was 7 mM, ruling out any role of these genes in enabling the strain to survive in the presence of the compound.

**Figure 3:**
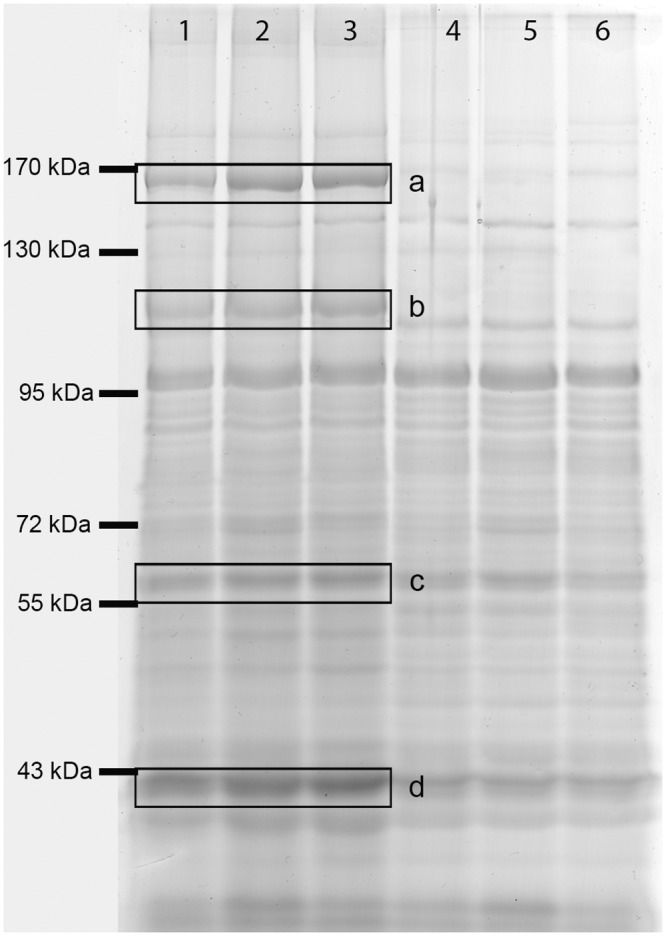
SDS-PAGE of the membrane fraction of *P. protegens* Pf-5 grown in presence and absence of FA. Triplicate cultures of *P. protegens* Pf-5 were grown aerobically for 12 h in E_2_ glucose minimal medium with (1, 2 and 3) or without (4, 5 and 6) the addition of 2 mM FA. Aliquots containing 100 μg of protein obtained from the total membrane fraction were loaded onto the gel. Boxed bands mark proteins whose levels differ in cells grown in the presence and absence of FA and were identified by peptide fingerprint analysis (a: Pyochelin synthetase F, b: Pyochelin synthetase E, c: ABC transporter ATP-binding protein, d: L-ornithine 5-monooxygenase PvdA).

In order to identify molecular mechanisms that contribute to the resistance to FA, the cytosolic and membrane proteomes of *P. protegens* Pf-5 after exposure to 2 mM FA for 12 h were compared to those of a control culture grown without FA. Analysis of the proteins of the cytosolic fraction did not reveal any significant differences (Figure in S1 File). However, comparison of the patterns in the membrane fractions of treated and untreated *P. protegens* Pf-5 cells revealed that the levels of several proteins was up-regulated in cells cultured in the presence of FA (Fig. 3). These proteins were identified by MALDI-TOF mass spectrometry, and found to correspond to proteins known or supposed to be involved in iron acquisition. Three of the proteins identified participate in the synthesis of siderophores. Pyochelin synthetase F and pyochelin synthetase E catalyze the final steps in the synthesis of enanto-pyochelin (EPch) [31], while L-ornithine monooxygenase acts in the synthesis of pyoverdine, the other siderophore secreted by *P. protegens* Pf-5 under iron-limiting conditions [39]. We also identified a transport protein of unknown function (encoded by the locus tag PFL_5344), which shows high similarity to an ABC-type transporter present in the mitochondrial membrane and is presumably involved in iron uptake into the organelle in eukaryotes. These results suggest a correlation between iron acquisition and resistance to FA in *P. protegens*.

### *P. protegens* mutants that are unable to produce siderophores are highly sensitive to FA

**Figure 4:**
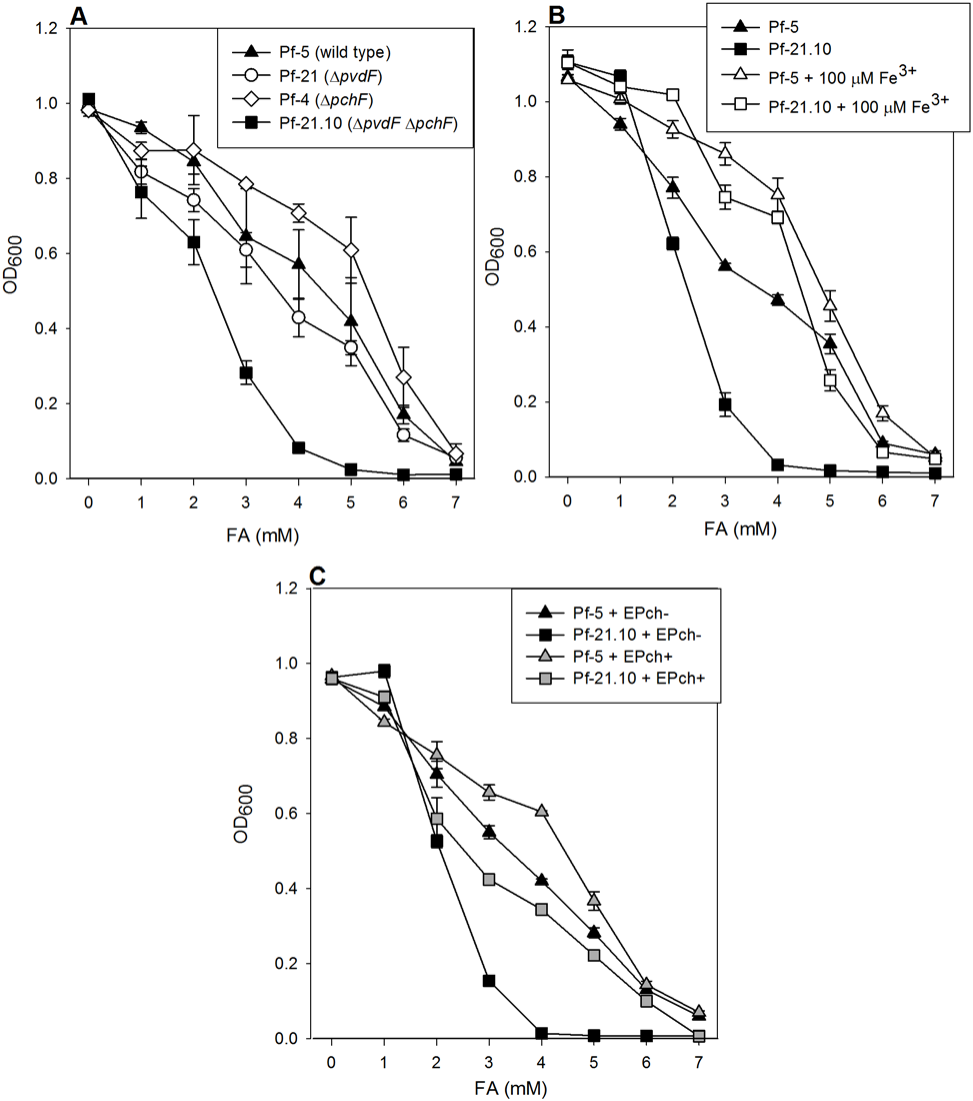
Correlation between siderophore production in *P. protegens* and resistance to FA. *P. protegens* Pf-5, *P. protegens* Pf-21, *P. protegens* Pf-4 and *P. protegens* Pf-21.10 were cultured aerobically during 16 h in 96-well polystyrene plates containing E_2_ glucose minimal medium supplemented with increasing concentrations of FA (**A**). Cultures of *P. protegens* Pf-5 (wild type) and *P. protegens* Pf-21.10 (Δ*pvdF*, Δ*pchF*) were grown under the same conditions with or without FeCl_3_ supplementation at a final concentration of 100 μM (**B**). Cultures of *P. protegens* Pf-5 (wild type) and *P. protegens* Pf-21.10 (Δ*pvdF*, Δ*pchF*) were grown under the same conditions with or without EPch supplementation at a final concentration of 1.3 μM (**C**). Values of maximal cell densities (OD_600_) are shown. The experiments were performed three times and error bars represent the standard deviation of the mean.

In order to evaluate the role of siderophore production in resistance to FA, deletion strains were constructed for the genes *pvdF* and *pchF*, which code for the last enzymes in the biosynthetic pathways leading to pyoverdine and EPch, respectively. Single deletion mutants in *pvdF* or *pchF* behaved essentially like the wild-type strain when grown in glucose E_2_ medium containing various concentrations of FA (Fig. 4A). In contrast, a double deletion mutant unable to produce either pyoverdine or EPch was notably less tolerant to FA (Fig. 4A). While the minimal inhibitory concentration in glucose minimal E_2_ medium for *P. protegens* Pf-5 was 7 mM, the double mutant *P. protegens* Pf-21.10 showed a MIC of 4 mM, and its ability to grow in the presence of FA was markedly reduced (Fig. 4A). The minimal E_2_ medium is routinely supplemented with a mixture of trace elements [22] containing 10 μM Fe^3+^, a concentration sufficient to support normal growth. To test whether FA causes iron limitation by chelating the metal, *P. protegens* Pf-5 and the double mutant *P. protegens* Pf-21.10 were grown in the presence of various concentrations of FA under conditions of iron excess (100 μM Fe^3+^) (Fig. 4B). Under these conditions, the wild type and the double deletion mutant showed comparable levels of resistance. Moreover, the wild type grew to higher densities in FA-containing medium that had been supplemented with 100 μM Fe^3+^ (Fig. 4B). These results further support the idea that FA limits cell growth by sequestering iron and making this nutrient unavailable to the cells.

As an additional test, we asked whether addition of purified EPch (1.3 μM) could enhance the resistance of the siderophore-negative strain *P. protegens* Pf-21.10 to FA. The MIC determined for the double mutant strain in the absence of EPch was 4 mM; in the presence of EPch a MIC of 7 mM was measured (Fig. 4C). Moreover, addition of EPch also enhanced the growth of *P. protegens* Pf-5 in medium containing 3 mM, 4 or 5 mM FA. These results demonstrate that the presence of EPch is enough to stimulate growth in the presence of FA.

### FA promotes secretion of pyoverdine

**Figure 5:**
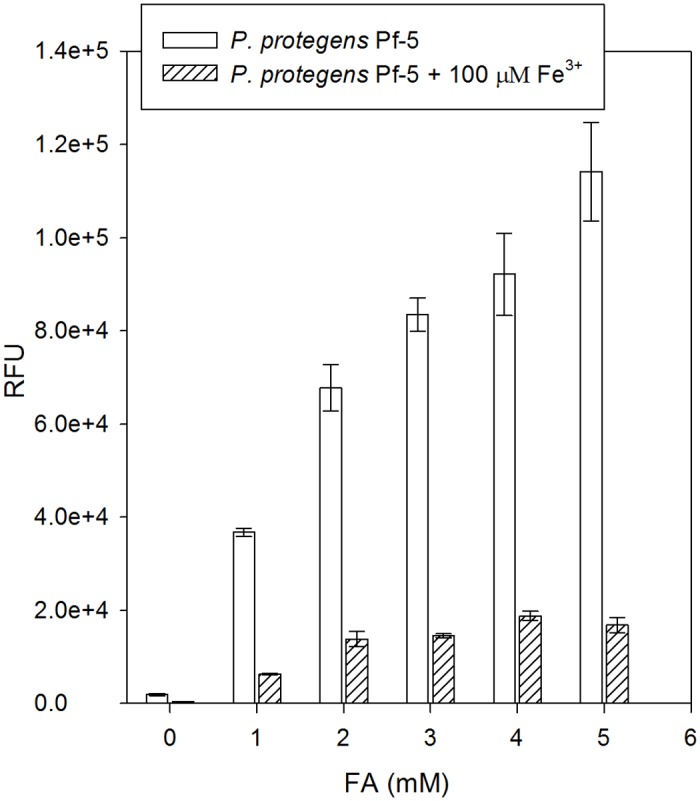
Effect of FA and iron supplementation on pyoverdine production. *P. protegens* Pf-5 was cultured aerobically during 16 h in 96-well polystyrene plates containing E_2_ glucose minimal medium with or without increasing concentrations of FA and with or without FeCl_3_ supplementation at a final concentration of 100 μM. The fluorescence intensity of the supernatant was measured with a fluorimeter and growth was estimated by determination of the OD_600_. The fluorescence intensity obtained was normalized to the OD_600_, and is thus expressed in relative fluorescence units (RFU). The experiments were performed three times and error bars represent the standard deviation of the mean.

To corroborate the correlation between FA and siderophores, we measured the pyoverdine content in the supernatant of an overnight culture of *P. protegens* Pf-5 by measuring the fluorescence emission as described in Materials and Methods. As a control of the method, the fluorescence emission in the supernatant of the mutant unable to produce pyoverdine, P. *protegens* Pf-21, was also evaluated. No fluorescence emission was observed in this strain. Fig. 5 clearly shows that the pyoverdine content in the supernatant of *P. protegens* Pf-5 grown with 10 μM Fe^3+^ increases with the concentration of FA added to the culture medium. The production of pyoverdine increased by about 19-fold when 1 mM FA was added to the culture medium and by almost 60-fold with 5 mM FA. Under conditions of iron excess (100 μM Fe^3+^), the pyoverdine content also rose with increasing concentrations of FA, but the overall fluorescence intensity was much lower.

### FA chelates Fe^3+^ and other metals

As a further test, we determined whether FA forms chelates with iron and other metals *in vitro* directly. The spectrophotometric method has been described previously for the estimation of chelates [40, 41, 42, 43]. Table 2 shows the affinity constant (K_0.5_) of FA for Fe^2+^, Fe^3+^, Cu^2+^, Mn^2+^ and Zn^2+^. As shown in Table 2, all K_0.5_ values obtained were in the same order of magnitude in the μM range, but Fe^3+^ was the metal that showed the highest affinity for FA. This result demonstrates that FA has iron-chelating properties, and sequesters metal ions from the medium. In agreement with this, we confirmed in a separate experiment, in which the FA concentration in the medium was measured, that FA is not taken up into the cell (Table in S1 File).

**Table 2:** Chelate formation of FA with different metals.

**Metal**	Apparent affinity constant (K_0.5_)*
Fe^2+^	55 μM
Fe^3+^	10 μM
Cu^2+^	22 μM
Zn^2+^	49 μM
Mn^2+^	86 μM

* The data represent the means of three determinations calculated as described in Materials and Methods.

## Discussion

Fusaric acid is a fungal metabolite produced by the genera *Fusarium* [44, 45], and was one of the first mycotoxins to be implicated in the pathogenesis of wilt disease in large numbers of plant species [44]. Besides being toxic to plants, FA is harmful to many bacteria [18, 46]. Previous work had demonstrated that rhizobacteria belonging to the genera *Bacillus* and *Paenibacillus* are much more susceptible to FA than fluorescent *Pseudomonas* spp. Moreover, different strains of fluorescent *Pseudomonas* spp. were found to vary widely in their sensitivity to FA [46]. Besides having a negative effect on growth, FA has also been shown to inhibit production of some secondary metabolites implicated in biocontrol of soil-borne diseases by various strains of fluorescent *Pseudomonas* spp. [12, 15, 16, 17].

The results presented in this work show that FA reduces growth rate, motility and viability during stationary phase, while increasing substrate attachment of the biocontrol strain *P. protegens* Pf-5 (Figs. 1 and 2). By reducing bacterial dispersion and favoring bacterial attachment to plant roots, this response could serve to enhance bacterial survival in the presence of the mycotoxin in the soil.

With the aim of understanding the mechanism of toxicity of FA and defining the responses it elicits in *P. protegens* Pf-5, we examined the protein composition of subcellular fractions obtained from cells grown in the absence and presence of the compound. The most striking difference detected was that some enzymes involved in the biosynthesis of the two major siderophores produced by *P. protegens* Pf-5, pyoverdine and enantio-pyochelin, were present at higher levels in the membrane fraction obtained from bacteria cultured in the presence of the toxin (Fig. 3). Moreover, production of pyoverdine was significantly increased when cells were grown in the presence of FA, and the magnitude of the increase was dependent on the concentration of FA added to the medium (Fig. 5). In agreement with our results, several genes implicated in pyoverdine biosynthesis and iron uptake in *P. chlororaphis* PCL1392 have been found to be up-regulated in the presence of FA [16]. A recent study of the transcriptional response to iron limitation in *P. protegens* Pf-5 had also revealed that genes involved in pyoverdine and enanto-pyochelin biosynthesis and uptake, as well as heme acquisition systems, were up-regulated under these conditions, while the expression of some genes involved in flagellar biosynthesis was repressed [47]. Phenotypic tests confirmed a reduction in swarming motility in response to iron limitation [47]. Taken together, these results reveal that FA induces a concentration dependent response and suggest that iron chelation is the main toxic effect of FA. Note that FA has also been shown to inhibit eukaryotic cell growth [48] [49], and a related compound, picolinic acid (pyridine-2-carboxylic acid) is known to inhibit iron incorporation in eukaryotes [50].

Spectrophotometric analyses confirm that FA can chelate metal cations such as Fe^2+^, Fe^3+^, Cu^2+^, Mn^2+^ and Zn^2+^ (Table 2). Of these, Fe^3+^ showed the highest affinity for FA. Several studies have demonstrated that minerals influence the production of antimicrobial compounds by fluorescent pseudomonads. Thus, the expression of genes involved in DAPG synthesis is known to be stimulated by Fe^2+^, Zn^2+^ and Cu^2+^ [47, 51, 52] and repressed by FA [12, 17]. The ability of FA to form chelates with these cations can therefore account for the negative impact of FA on DAPG production.

To evaluate the role of pyoverdine and EPch during growth of *P. protegens* Pf-5 in the presence of FA, single and double mutants lacking genes for the enzymes that carry out the final steps in the synthesis of these siderophores were generated. The double mutant showed a notably reduced ability to cope with FA (Fig. 4). This growth disadvantage was eliminated when the medium was supplemented with an excess of iron or contained the purified EPch (Fig. 4B and 4C). Pyoverdine is a complex water-soluble chelator of ferric iron (approximately 1,500 Da), which binds the metal with a stoichiometry of 1:1 [53] and an affinity constant of approximately 10^24^ M^-1^ [39, 53]. In contrast, EPch [31], the enantiomer of the siderophore pyochelin produced by *P. aeruginosa* [54, 55], is a poorly water-soluble compound of low molecular weight (325 Da), which binds ferric iron with a stoichiometry of two molecules per iron atom and a much lower affinity constant of approximately 2 × 10^5^ M^-1^ [32]. It is important to note that the affinity of pyochelin for ferric iron is comparable to that calculated for the binding of FA to the cation (Table 2). It is therefore reasonable to expect that EPch is able to compete with FA for the available iron.

According to the data obtained in this study, at least one class of siderophore is needed to support growth of *P. protegens* Pf-5 in the presence of FA and absence of excess iron. Several studies have demonstrated the importance of siderophores in the suppression of wilt diseases caused by FA-producing *Fusarium* spp. [56, 57, 58]. Considering that soil is an iron-limited environment, the results obtained in this work provide an additional explanation for this effect. The fact that the addition of a low level of EPch can enhance the growth of *P. protegens* in the presence of FA underlines the role of this siderophore in the biocontrol of *Fusarium* spp.

In summary, the results presented in this work suggest that the mycotoxin FA exerts its toxic effect on *P. protegens* Pf-5 mainly by sequestering iron. As a result of iron chelation by FA, siderophore synthesis is induced. As FA is also able to chelate other metals (Table 2) and Fe^3+^ is not the only metal cation known to regulate the production of pyochelin in *P. aeruginosa* [59], EPch synthesis in *P. protegens* may be induced by deprivation of metals other than iron. At all events, the ability of *P. protegens* Pf-5 to produce siderophores markedly increases its resistance to FA, suggesting that this capacity would confer a clear adaptive advantage in soils in which FA-producing *Fusarium* spp. are present. These results highlight the importance of using biocontrol strains that excrete elevated levels of siderophores in agricultural systems inhabited by FA producers and also emphasize the relevance of iron supplementation to improve the biocontrol of *Fusarium* wilt.

## Supporting Information

S1 File Figure: Two-dimensional gel electrophoresis of the cytosolic fraction from *P. protegens* Pf-5 cells grown in presence (a) and absence (b) of 2 mM FA. Proteins were separated by IEF on a linear pH gradient from 3 to 10 and on 13% SDS-PAGE gels and stained with Coomasie brilliant blue. **Table: Measurement of FA in the supernatant of cultures of *P. protegens* Pf-5 and *E. coli* MG1655**.
(PDF)
