# Supplementary material for: Production of Siderophores Increases Resistance to Fusaric Acid in Pseudomonas protegens Pf-5
Source: PLoS One. 2015 Jan 8;10(1):e0117040. doi: 10.1371/journal.pone.0117040 (PMC4287623; doi:10.1371/journal.pone.0117040)
Supplement: S1 File — Proteins were separated by IEF on a linear pH gradient from 3 to 10 and on 13% SDS-PAGE gels and stained with Coomasie brilliant blue. Table: Measurement of FA in the supernatant of cultures of P. protegens Pf-5 and E. coli MG1655. (PDF) [file pone.0117040.s001.pdf]

Supporting Information

Production of siderophores increases resistance to fusaric acid in *Pseudomonas protegens* Pf-5

Jimena A. Ruiz[1,2,3]*, Evangelina M. Bernar[3,4] and Kirsten Jung[2*]

[1]Instituto de Investigaciones en Biociencias Agrícolas y Ambientales, CONICET, Facultad de Agronomía, Universidad de Buenos Aires. Avenida San Martín 4453, C1417DSE Ciudad Autónoma de Buenos Aires, Argentina

[2]Ludwig-Maximilians-Universität München, Munich Center for integrated Protein Science (CiPSM) at the Department of Biology I, Microbiology, Großhaderner Straße 2-4, 82152 Martinsried, Germany

[3]Departamento de Química Biológica, Facultad de Ciencias Exactas y Naturales, Universidad de Buenos Aires. Intendente Güiraldes 2160, C1428EGA Ciudad Autónoma de Buenos Aires, Argentina

[4]Current address: Departamento de Química Biológica e Instituto de Bioquímica y Biofísica (IQUIFIB, UBA-CONICET), Facultad de Farmacia y Bioquímica, Universidad de Buenos Aires. Junín 956, C1113AAD Ciudad Autónoma de Buenos Aires, Argentina.

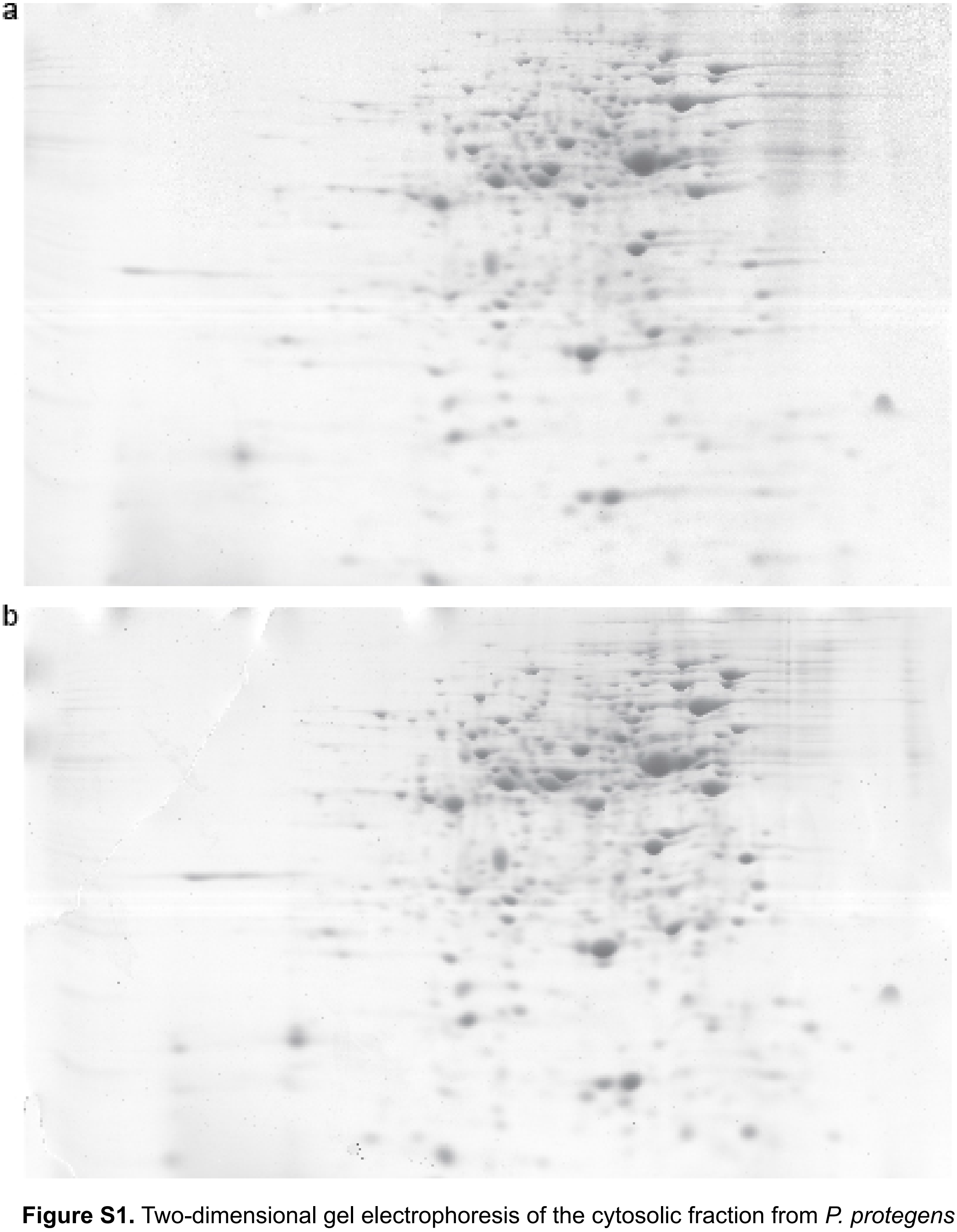

Figure S1. Two-dimensional gel electrophoresis of the cytosolic fraction from *P. protegens* Pf-5 cells grown in presence (a) and absence (b) of 2 mM FA.

Table S1. Measurement of FA in the supernatant of cultures of *P. protegens* Pf-5 and *E. coli* MG1655

Supernatant or medium	FA (mM)*
**Controls**	
E_2 medium + 0.1 mM FA	0.0547± 0.00072
E_2 medium without MT + 0.1 mM FA	0.0909 ± 0.00099
**Supernatants**	
P. protegens Pf-5	0.0521 ± 0.0011
E. coli MG1655	0.0514 ± 0.00097

* The data represent the mean of three determinations measured by HPLC as described in Materials and Methods.
